# On the Use of Sulphate of Bebeerine as a Substitute for Sulphate of Quinia

**Published:** 1853-09

**Authors:** Edward D. Dailey

**Affiliations:** Smyrna, Del.


### *On the use of Sulphate of Bebeerine as a substitute for Sulphate of Quinia.* By Edward D. Dailey, M. D., of Smyrna, Del.

My attention was first called to the use of the *Sulphate of Bebeerine* as a substitute for quinia, in remittent and intermittent fevers, by the note of Prof. H. S. Patterson, published in the "Medical Examiner" for May, 1852, in which he urged upon the profession, the propriety of giving that drug a fair and full trial, both on account of its comparative cheapness and its freedom from producing the unpleasant effect upon the brain which sometimes follows the administration of quinine. Hoping I had found a remedy suited, in a great measure, to take the place of the last named salt, equally and in some cases more efficacious, and not obnoxious to the objection that it (quinine) is on account of its price, I sent to Philadelphia immediately—having at the time several obstinate cases of intermittent under treatment—and procured a quantity, for the purpose of giving the bebeerine a trial, and in which cases its anti-periodic properties were fully tested.

Case 1st.—Mrs. H——, a widow lady from Philadelphia, then on a visit to Hunting Creek, Md.,—where I at that time resided—had had an intermittent for several months, for which quinia, bark in substance, the various preparations if iron, zinc, &c., had been used in succession, with the effect of checking the chill for a few days only ; she had almost concluded to abandon entirely the use of medicine, and I was at a loss what to do for her (she postively refused to take arsenic) until I received the bebeerine. I then commenced giving her this in accordance with the recommendation of Prof. P., viz., ʒss. to ℥viii. water, with enough Elix. Vitriol to dissolve it, of which a table spoonful was taken every third hour during the apyrexia. The next paroxysm was not prevented but was delayed nearly three hours. After the subsidence of fever, she again took the bebeerine in the same manner, and so continued for two days entirely escaping the expected chill, nor had she any return during her stay in Maryland, which was five months after "missing her chill." I, however, continued the administration of the remedy by way of precaution, and on account of its tonic properties, gradually diminishing the dose, and at longer intervals, for a week. With the result of this trial, I was highly gratified, and was confirmed in the determination to still further test its power.

Case II.—Miss H. D——, aged 17, had had three fits of ague, recurring every day, one day in the morning, the next in the afternoon—a double tertian. The patient was of bilious temperament, tongue covered with a dirty white coating, complexion icterode, sclerotica in the same condition, bowels constipated, with scanty high colored urine. I gave hydrarg. proto chlor. grs. x. to be followed in six hours by Ol. ricini ℥j., which purged her freely. I then gave the bebeerine in the same manner as in case I. Still the paroxysms continued to occur with no sign of abatement, notwithstanding I persisted in the treatment until she complained of a burning sensation in the stomach, when I withdrew the bebeerine, and one hour previous to the time for the next paroxysm, gave her sulph. quinine grs. viii., which completely warded off the chill; and by the use of Vallet's proto-carb. iron she speedily regained her strength and color.

Since the above I have used it in many cases with variable success, but as a general rule I have not been so well satisfied with its results as I have been with those from quinia. Notwithstanding I have used four ounces I do not know that I have met with more than three cases in which the recovery was clearly attributable to the bebeerine.

That the sulphate of bebeerine will ever supersede the sulph. of quinine I have not the remotest idea; first, because its *anti-periodic* properties are not so certain nor so speedily obtained; 2dly, because the sulph. of quinia is by far the cheaper article of the two.

Quinine may be had for from three dollars to four and a half per ounce, and from ten to twenty grains will usually cure an intermittent; whilst on the other hand, I paid for bebeerine fifty cents a drachm, which brings it above the average price of quinine, and in no case will less than from 30 to 60 grains effect a cure.

That bebeerine is a good general tonic, no one who has used it, will, I apprehend deny, and for that purpose I am in the habit of prescribing it; but even as a tonic we have others equally efficacious and much lower in price. At my suggestion, my friend Dr. W. D. Noble, of Federalsburg, Md., and my brother

Dr. S. T. Dailey of Somerset county, in the same State, have used bebeerine and their experience corresponds with my own.

